# NMR Relaxometry Across Time: From Early Insights to Emerging Directions

**DOI:** 10.1002/mrc.70002

**Published:** 2025-06-19

**Authors:** Pellegrino Conte, David Faux, Anne‐Laure Rollet, Delia Chillura Martino, Danuta Kruk, Gianni Ferrante, Paolo Lo Meo

**Affiliations:** ^1^ Department of Agriculture, Food and Forest Sciences University of Palermo Palermo Italy; ^2^ Physics Department University of Surrey Surrey UK; ^3^ Laboratoire PHENIX (UMR 8234‐CNRS/UPMC) Paris France; ^4^ Department of Biological, Chemical and Pharmaceutical Sciences and Technologies University of Palermo Palermo Italy; ^5^ Department of Physics and Biophysics University of Warmia and Mazury in Olsztyn Olsztyn Poland; ^6^ Stelar Srl., Via Enrico Fermi 4 Mede Italy

**Keywords:** fast field‐cycling NMR, molecular dynamics, multimodal characterization, NMR relaxometry, nuclear magnetic resonance dispersion (NMRD), time‐domain NMR

## Abstract

Nuclear magnetic resonance (NMR) relaxometry has evolved from early theoretical insights into a dynamic and versatile analytical technique capable of probing molecular and ionic motion across diverse fields. Rooted in the foundational work by many different scientists (e.g., Bloch, Purcell, Torrey, Hahn, Bloembergen, Pound, and Solomon, just to name a few), relaxometry has progressed through pivotal advancements such as Redfield's theory and the development of time‐domain (TD) and fast field‐cycling (FFC) methodologies. While the former enables rapid, low‐cost analysis of relaxation time distributions, widely applied in soft matter and quality control, the latter provides frequency‐resolved nuclear magnetic resonance dispersion (NMRD) profiles that capture dynamic processes across multiple timescales, revealing deeper insights into molecular interactions in heterogeneous systems. Recent innovations in instrumentation have expanded the applicability of relaxometry. Moreover, its integration with modalities such as diffusimetry and imaging has opened new routes for spatially resolved and multimodal analyses. Applications now span materials science, biomedicine, and environmental studies. In polymers and porous media, relaxometry reveals segmental dynamics and surface interactions; in biological tissues, NMRD profiles differentiate healthy from pathological states, offering diagnostic potential. Emerging applications include contrast agent development, soil hydration analysis, microplastic detection, and wastewater monitoring. This paper offers a comprehensive overview of the field's historical trajectory, methodological advancements, and expanding application landscape. Emphasis is placed on the synergy between TD and FFC‐NMR approaches and the ongoing transition toward portable, real‐time, and multimodal relaxometric systems. NMR relaxometry is poised to become a mainstream tool in diagnostics, materials characterization, and environmental monitoring.

## Graphical abstract

NMR relaxometry bridges time and frequency domains to probe molecular dynamics across materials, biomedical, and environmental systems. This perspective highlights advances in time‐domain and fast field‐cycling methods, their synergy, and emerging applications enabled by miniaturized instrumentation, multimodal integration, and AI‐enhanced analysis.

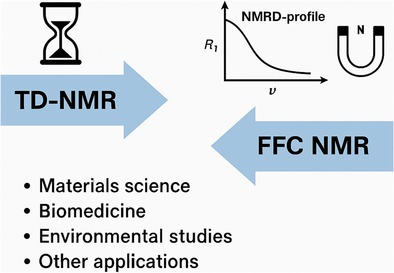

## 1 Introduction

Nuclear magnetic resonance (NMR) relaxometry—the study of nuclear spin relaxation as a probe of molecular dynamics—traces its roots to the earliest NMR experiments. The foundational discoveries by Bloch et al. [1] and Purcell et al. [2] in 1946 established the basis for magnetic resonance, where the longitudinal (T_1_) and transverse (T_2_) relaxation times became central observables for probing molecular motion and environmental interactions.

**FIGURE 1:**
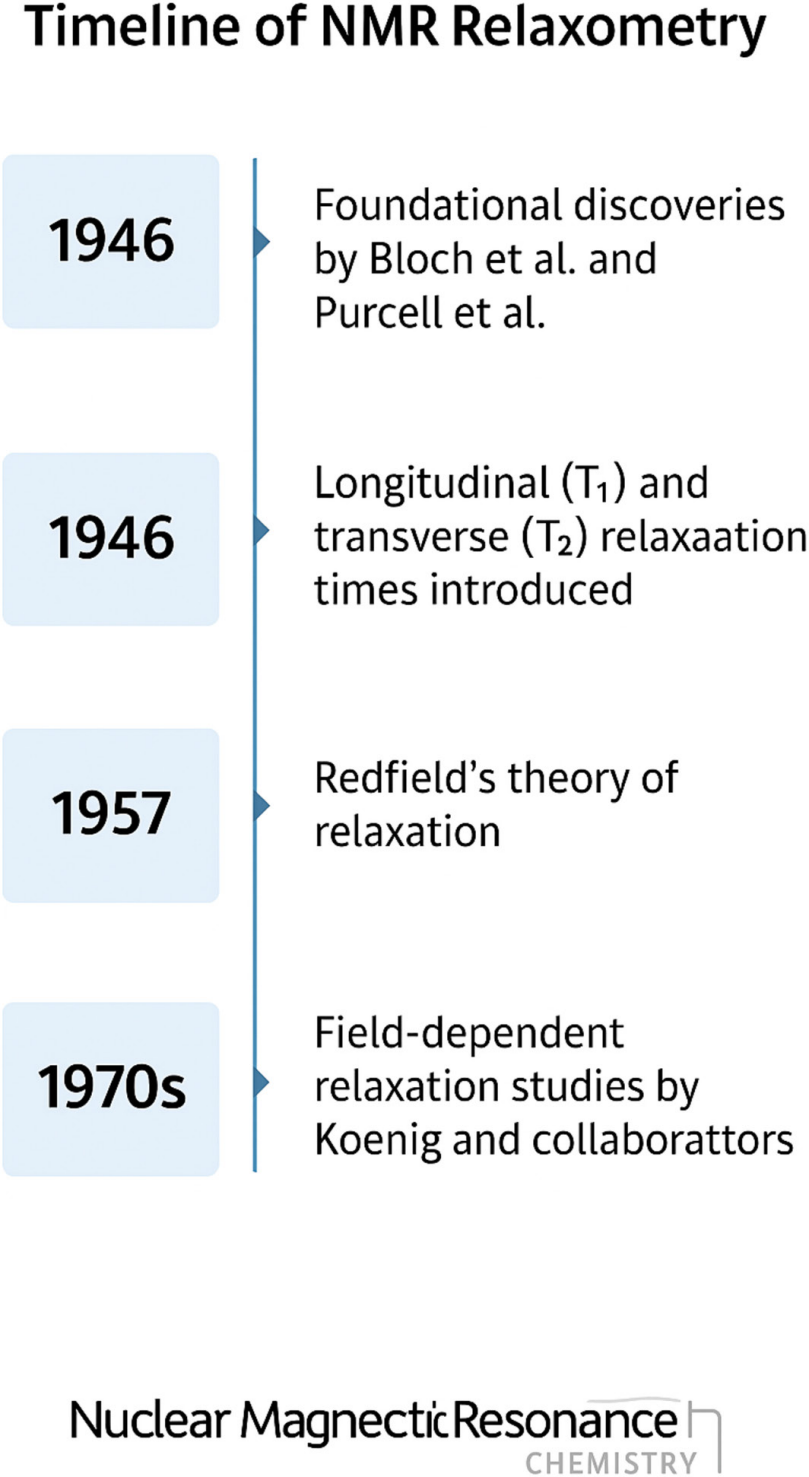
Timeline of relaxometry development.

In subsequent decades (Figure 1), many theoretical developments have emerged, ranging from the famous Bloembergen, Purcell, and Pound (BPP) model [3], through the Solomon equations [4], to Redfield's theory [5]. Other pioneering experimental studies, such as that by Koenig and collaborators [6] in the 1970s, laid the groundwork for field‐dependent relaxation studies, particularly in biological systems.

The modern concept of NMR relaxometry—encompassing both time‐domain (TD) NMR and fast field‐cycling (FFC) NMR—emerged in the 1980s as a way to nondestructively characterize complex materials and biological tissues by quantifying how nuclear spin relaxation varies with magnetic field or time. TD‐NMR operates by applying a magnetic field to a sample, measuring the time it takes for the nuclear magnetization to return to equilibrium after perturbation, and providing a relaxation time distribution (either T_1_ or T_2_). Since relaxation time values are directly related to molecular dynamics, the relaxation time distribution provides direct insight into the various types of molecular motions, such as diffusive or rotational [7, 8]. This distribution is analyzed applying simple bi‐exponential fits [9] or using more sophisticated transforms [10, 11, 12, 13, 14, 15, 16] to reveal the heterogeneity of molecular dynamics in the system.

**FIGURE 2:**
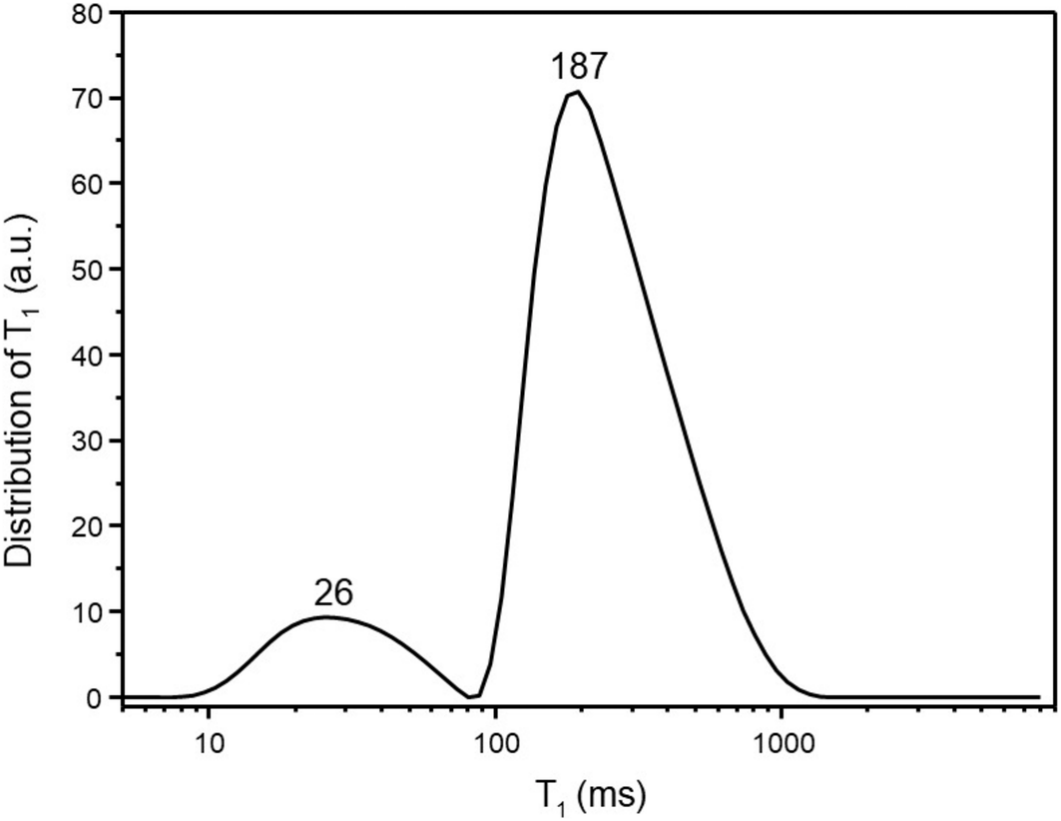
Typical TD‐NMR profile (i.e., distribution of T_1_) for a biochar obtained using a Kon Tiki kiln system. The pyrolysis quenching was performed by adding dry soil on top of the kiln and allowing the temperature to gradually decrease to room temperature. The sample was prepared by adding deionized water to the biochar in a 3:1 (water‐to‐biochar) ratio. The proton Larmor frequency applied in this experiment was 35 MHz. This T_1_ distribution has not been previously published and is part of Conte's database.

As a general remark, relaxometry is widely used for liquids, biological tissues, and polymeric materials, providing valuable insights into molecular mobility, molecular interactions, and viscosity [17]. More recently, TD‐NMR has also been applied to evaluate environmentally relevant samples. For example, Figure 2 reports a typical TD‐NMR profile for a biochar obtained by Kon Tiki kiln [18] showing two distinct components corresponding to more immobilized water on the biochar surface (T_1_ = 187 ms) and bulk water (T_1_ = 26 ms).

**FIGURE 3:**
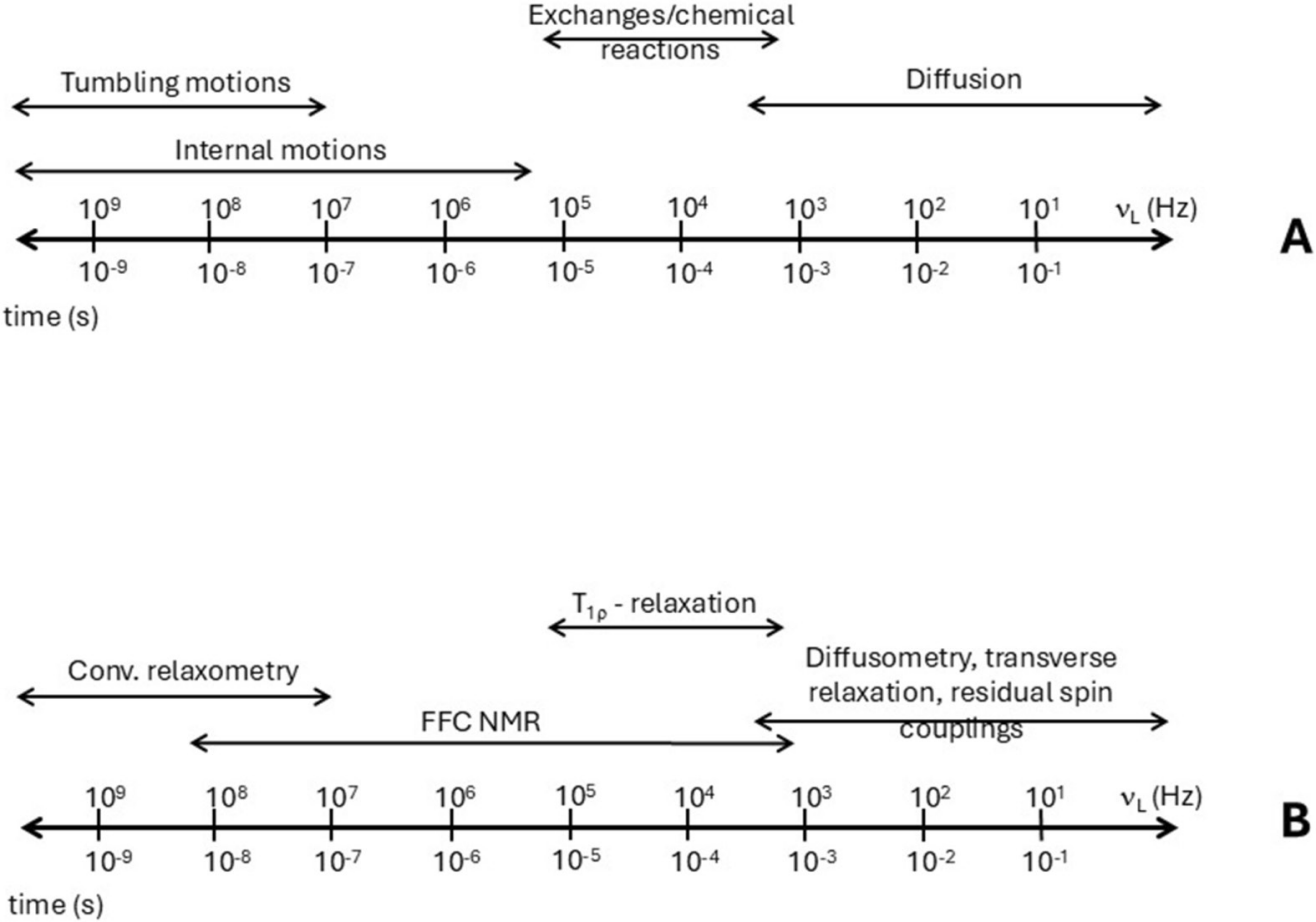
(**A**) Molecular dynamics timescales probed by experimental techniques. Tumbling refers to rotational diffusion of molecules or molecular segments. Internal motions mean localized movements including: (i) fast dynamics, such as aromatic ring oscillations, and aliphatic chain flexing; (ii) slow rearrangements, such as hydrophobic domain fluctuations and hydrogen‐bonding network shifts. Diffusion refers to translational displacement of molecules. (**B**) NMR techniques for monitoring these motional regimes.

With instrumental advancements, FFC‐NMR emerged as a further complementary technique, allowing for the measurement of relaxation times across a range of magnetic field strengths [17]. Unlike TD‐NMR, which relies on a single field, FFC‐NMR uses a field‐cycling approach, where the sample is exposed to a varying magnetic field during the experiment, thus providing a more detailed multidimensional view of molecular dynamics [17, 19, 20]. While TD‐NMR has found broad application in industrial and soft matter analysis, FFC‐NMR uniquely enables the acquisition of nuclear magnetic resonance dispersion (NMRD) profiles, providing deeper insight into molecular dynamics across multiple timescales (Figure 3). Figure 3A illustrates the characteristic frequency ranges of molecular motions probed by NMR, while Figure 3B presents the corresponding NMR techniques commonly employed to monitor these different dynamic processes.

Over the last decades, the field has witnessed significant developments. On one hand, new algorithmic approaches, such as the uniform penalty inversion of multiexponential decay [21, 22, 23, 24], the L1‐regularized data inversion [15, 25], and the heuristic algorithm for the analysis of the magnetization curves, have been proposed [26], and on the other hand, new software implementing different dynamical models are available, such as the model‐free analyses [27, 28], the 3‐Tau model [29], and Fitteia [30], that have facilitated NMRD data interpretation. Applications have expanded from polymeric membranes [31] to biomedical diagnostics and dynamic contrast agents [32]. The launch of the European projects FC‐RELAX [33] and NMR‐IMPROV [33] underscores the growing institutional investment in exploiting NMR relaxometry for both materials science and medical imaging innovation.

This paper provides a critical overview of NMR relaxometry “across time”—from its theoretical and experimental origins to current technological breakthroughs—highlighting the interplay between TD and FFC approaches, emerging applications, and the future challenges that will shape the next generation of relaxometric methods.

## 2 What Is NMR Relaxometry

NMR relaxometry relies on measuring nuclear spin relaxation times—longitudinal (T_1_) or transverse (T_2_)—to gain insight into molecular motion and interactions with the surrounding environment. Relaxation arises from the fluctuating magnetic fields induced by molecular dynamics, which modulate the local magnetic environment experienced by nuclear spins. From a mathematical standpoint, the way the magnetic field fluctuates at different frequencies and timescales is captured by the spectral density function, *J*(ω), which describes how these fluctuations depend on frequency—namely, the Fourier transform of the self‐correlation function of the nucleus motion [1, 2, 6, 17, 34]. Hence, there is a direct link between molecular motion and the relaxation times. More precisely, the spin–lattice relaxation (measured by *T*_*1*_) of dipolar nuclei, such as ^1^H, depends on *J*(ω) and *J*(2ω). Accordingly, T_1_ captures the dynamics near the Larmor frequency ω, i.e., roughly in a time window around 1/(2πω). This is a key point in understanding the key advantage of the FFC technique in the study of dynamics in complex media. Indeed, it shows that at high field, T_1_ reveals the fast motion like molecular rotation, whereas at low field, T_1_ reveals the slow motion like translational diffusion, diffusion on surface, etc.

**FIGURE 4:**
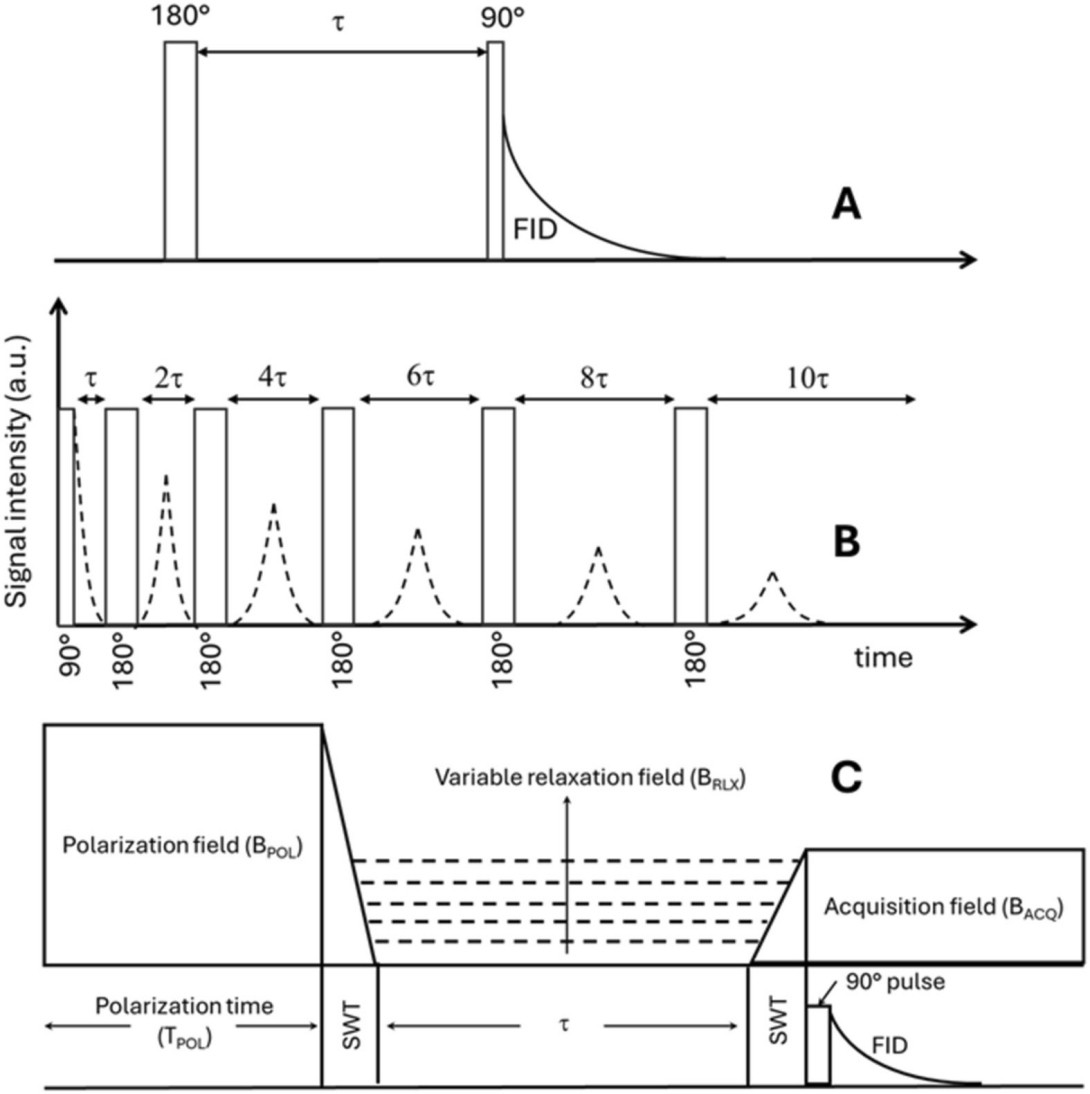
(**A**) Inversion recovery pulse sequence to measure the T_1_ value at fixed proton Larmor frequency. (**B**) CPMG pulse sequence to measure the T_2_ value of a molecular system at fixed proton Larmor frequency. (**C**) Typical FFC‐NMR relaxometry sequence.

The spin–spin relaxation (T_2_), related to the dephasing of the transverse magnetization, depends on *J*(ω = 0), *J*(ω), and *J*(2ω). The term *J*(ω = 0) often dominates, making T_2_ a more sensitive probe in TD‐NMR experiments than T_1_ for the dynamics near interfaces. However, TD‐NMR gives access to only two points in the spectral density with T_1_ and T_2_, whereas by analyzing the whole spectral density, NMR relaxometry reveals important information about molecular motion, including rotational and diffusive processes, and helps assess the viscoelastic properties of the medium. This function is integral to the interpretation of relaxation times and the study of materials, from liquids to biological tissues, offering a deeper understanding of molecular interactions and material behavior. The measurement of relaxation times differs between FFC and TD‐NMR. In particular, FFC‐NMR technique operates by rapidly switching the magnetic field during a pulse sequence: The sample is polarized at a high field, relaxed at a variable evolution field, and detected at a separate detection field (Figure 4C). Namely, the application of a polarization field, B_POL_, occurs for a fixed period of time, referred to as polarization time, T_POL_. This time can either be non‐null (resulting in a prepolarized, PP, sequence) or null (yielding a nonpolarized, NP, sequence). The prepolarization is necessary to generate the magnetization, which then evolves to reach a new equilibrium under the influence of a relaxation field (B_RLX_) applied for a variable period of time indicated as 𝜏. After this period, the magnetic field intensity is switched to a new value (indicated as B_ACQ_), applied for another fixed period of time, T_ACQ_, while a 90° pulse is used to generate the observable magnetization. During the acquisition time, and after the application of the 90° pulse, the free induction decay (FID) is finally acquired. It is worth noting that the acquisition field, B_ACQ_, is needed to keep the magnetization intensity during the acquisition. The use of the PP sequence is recommended whenever the intensity of the relaxation field is very low; therefore, enhancement in sensitivity is needed. The crossover field between the PP and the NP sequences is empirically fixed at $\omega_{\mathrm{RLX}}=\omega_{\mathrm{POL}}/2$, where $\omega_{\mathrm{RLX}}$ and $\omega_{\mathrm{POL}}$ are the B_RLX_ and the B_POL_ proton Larmor frequencies expressed in MHz, respectively. A switching time (SWT) is also indicated. This is the time needed to switch between different magnetic field intensity values (Figure 4C).

TD‐NMR relaxometry, instead, typically uses low‐field instruments equipped with permanent magnets and standard pulse sequences. Both in high‐field NMR (typically with proton Larmor frequencies > 400 MHz) and in TD‐NMR, T_1_ and T_2_ relaxation times are measured using the inversion recovery and Carr‐Purcell‐Meiboom‐Gill (CPMG) pulse sequences, respectively (Figure 4A,B). The inversion recovery pulse sequence is 180°‐τ‐90°, where the first 180° pulse aligns the magnetization along the ‐z axis; then, after a variable time τ, the second 90° pulse generates the observable, and the FID is acquired. The signal intensity follows typically an exponential trend from which T_1_ is obtained (Figure 4A).

In the CPMG method, a 180° pulse train, where each pulse is separated by a given time τ, follows the application of the 90° pulse (Figure 4B). Echoes are then observed (the dotted curves in Figure 4B). The echo decay also typically follows an exponential law from which T_2_ can be retrieved. The robustness, low cost, and speed of acquisition of TD‐NMR mean that this technique is widely used in industrial and quality control applications.

Recent years have seen a growing interest in the combination of TD and FFC approaches. For instance, efforts have been made to bridge these domains by developing portable FFC‐NMR systems with improved switching speeds and increased field stability. Advances in data processing, such as inverse Laplace transforms and Bayesian reconstructions of relaxation distributions, have also enhanced the ability to resolve complex multicomponent relaxation behavior [25]. Moreover, recent studies have explored the synergy between NMR relaxometry and diffusimetry, integrating relaxation and self‐diffusion data to yield a richer description of confined or heterogeneous systems [31, 35]. Such approaches are particularly promising for porous media, soft matter, and tissues, where the interplay of molecular motion and structural heterogeneity plays a crucial role.

## 3 Instrumentation and Technical Advances

The development of NMR relaxometry has been closely tied to advances in instrumentation, particularly in the case of FFC systems. Traditional FFC relaxometers required bulky electromagnets and were limited by long switching times, low magnetic field stability, and insufficient sensitivity. Over the past 20 years, however, significant strides have been made in optimizing both hardware and control electronics. Current‐generation FFC devices can switch magnetic fields within milliseconds, enabling the acquisition of high‐resolution NMRD profiles with improved signal‐to‐noise ratios [36].

One of the key breakthroughs in recent years has been the miniaturization and improved integration of FFC hardware, opening pathways to benchtop or even portable FFC relaxometers for in situ analysis [17, 37, 38, 39]. Commercial instruments now allow routine scanning of fields from the microtesla to the tesla range with programmable polarization, evolution, and detection fields. In parallel, stabilization protocols and shielding improvements have reduced artifacts due to eddy currents and mechanical vibrations, allowing more precise relaxation measurements even at low fields [17].

For TD relaxometry, instrument design has focused on high‐throughput and automation for industrial and process‐monitoring environments. Innovations include arrayed coil geometries, parallel acquisition schemes, and nonconventional magnet configurations, including Halbach arrays and single‐sided magnets, which permit noninvasive characterization of macroscopic objects and layered materials [40].

Beyond hardware, signal processing and data analysis have emerged as crucial areas of innovation. Inversion of multiexponential decay curves—historically an ill‐posed problem—has been improved through regularized inverse Laplace transforms, Bayesian methods, and machine learning‐based fitting routines [25, 28, 30]. These techniques allow the extraction of relaxation time distributions and component‐resolved analysis, which is especially valuable in heterogeneous systems.

Another important technical development involves the combination of relaxometry with other magnetic resonance modalities. For example, integrated diffusion–relaxometry approaches [35] and relaxometry–imaging protocols are being explored for spatially resolved studies of tissues and porous materials [32, 39, 41]. These multimodal setups, though still largely in the research phase, represent a powerful step toward a more comprehensive and interpretable NMR‐based molecular characterization.

## 4 Applications in Materials Science

NMR relaxometry has established itself as a valuable tool in the characterization of materials, particularly those exhibiting structural heterogeneity or dynamic complexity. Its sensitivity to molecular motion across a wide range of timescales makes it uniquely suited for probing porous systems, polymers, ionic conductors, and soft condensed matter.

In porous materials, both TD and FFC relaxometry have been employed to investigate pore size distributions, surface interactions, and molecular mobility within confined spaces. The ability of FFC‐NMR to acquire NMRD profiles allows for the identification of slow dynamic processes and interactions at solid–liquid interfaces, which are not accessible by conventional high‐field NMR [17]. These profiles provide fingerprints of the interplay between diffusion, surface relaxation, and fluid–matrix interactions, and have been applied to the study of rocks, cementitious materials, and nanostructured solids [42, 43, 44, 45].

Worldwide, cement production accounts for approximately 8% of all carbon dioxide emissions and 7% of industry energy use [46]. Understanding the fundamental physics and chemistry of cement precarbonation, cement hydration, and the subsequent evolution of the cement paste is critical to developing products with a lower carbon footprint. Early applications of FFC‐NMR to cementitious materials led to the development of the Korb model, suitable for systems with NMRD profiles dominated by the interaction of mobile water with the electronic spins of fixed paramagnetic iron (III) [44]. Korb's model was later improved upon by the 3‐Tau model [29, 47, 48]. As a result, FFC‐NMR can now monitor changes in hydration chemistry with additives [49] and track the evolution of cement paste, providing insights into the development of durable, lower‐carbon cementitious products [50]. Future work will see important large‐scale properties such as compressive strength linked to the nanoscale evolution of the material.

In the domain of polymers and ionomers, relaxometry offers insight into segmental mobility, chain rigidity, and local ordering. A recent study employed FFC‐NMR to characterize the dynamic behavior of perfluorosulfonic acid membranes, demonstrating its potential in elucidating polymer dynamics relevant to fuel cell technology [31]. By comparing the dispersion of T_1_ values across frequency ranges, researchers were able to correlate hydration‐dependent proton mobility with polymer segment dynamics, contributing to the design of better‐performing materials [43].

Another key area is materials aging and degradation, where relaxometry can monitor changes in molecular mobility as indicators of structural fatigue, crosslinking, or thermal stress [51, 52, 53]. Noninvasive relaxometric measurements are especially useful for in situ monitoring of elastomers, coatings, and packaging materials in industrial environments [54].

Furthermore, advances in multimodal characterization have promoted the integration of relaxometry with techniques like magnetic resonance imaging (MRI), dielectric spectroscopy, and mechanical testing [55]. Such combinations enable correlative studies where molecular mobility, macroscopic mechanical properties, and microstructural features can be jointly analyzed [56]. Finally, the implementation of relaxometric techniques in one‐sided portable instrumentation, such as mobile universal surface explorer (MOUSE) or gradient at right angle to the field (GARfield), has provided a versatile and powerful tool for cultural heritage monitoring and preservation [57, 58].

In short, NMR relaxometry is increasingly recognized as a powerful method for structure–dynamics–function relationships in advanced materials, with applications ranging from industrial process control to the rational design of smart and responsive materials. Future research will aim to correlate large‐scale properties, such as compressive strength, with nanoscale structural evolution.

## 5 Applications in Biology and Medicine

NMR relaxometry, particularly in its FFC implementation, has emerged as a powerful tool for probing biological tissues and physiological fluids. Its ability to characterize molecular motion and exchange at multiple timescales offers unique insights that complement conventional high‐field NMR and MRI techniques.

A central application in this field is the characterization of tissue microstructure. The T_1_ dispersion curves (NMRD profiles) obtained via FFC‐NMR have been shown to differentiate between healthy and pathological tissues based on water mobility, protein interactions, and macromolecular crowding [59]. In particular, studies have demonstrated how relaxation dispersion patterns can serve as potential biomarkers for tissue composition and disease state, including cancer and neurodegeneration [39].

Additionally, FFC‐NMR has been explored for contrast agent design and optimization [60, 61]. Unlike conventional agents evaluated at fixed fields, FFC relaxometry enables the full characterization of relaxation processes across multiple magnetic field strengths, offering a pathway to develop field‐tunable or multimodal contrast agents [62, 63]. This approach supports the rational engineering of paramagnetic compounds with optimized efficacy at clinical field strengths or for ultra‐low‐field imaging technologies.

TD‐NMR has also been applied to biofluids and soft biological tissues, providing noninvasive assessments of hydration, viscosity, and macromolecular content. Such techniques are increasingly used in preclinical and clinical research for evaluating cartilage degeneration, liver fibrosis, and wound healing [64, 65].

One particularly promising area is the integration of FFC‐NMR relaxometry with low‐field MRI, where recent advancements have enabled the spatial mapping of relaxation dispersion. These hybrid techniques could enhance diagnostic accuracy in situations where conventional MRI lacks sensitivity, such as early detection of degenerative changes in cartilage or tumor tissue heterogeneity [66].

Despite these advances, challenges remain: The need for specialized instrumentation, low sensitivity at ultra‐low fields, and the complexity of data interpretation have limited clinical translation. However, initiatives like the FC‐RELAX [33] or NMR‐IMPROV [33] projects are actively addressing these barriers, aiming to bridge the gap between experimental relaxometry and practical biomedical applications.

## 6 Emerging Applications and Perspectives

NMR relaxometry continues to evolve, with emerging applications and methodological innovations expanding its role well beyond its traditional domains. The integration of novel acquisition strategies, miniaturized hardware, and hybrid measurement modalities is enabling access to previously unexplored systems and dynamic regimes.

One of the most active frontiers is the development of field‐tunable contrast agents and low‐field imaging technologies for biomedical use. The wide magnetic field range accessible through FFC‐NMR is essential for designing next‐generation agents tailored for specific field strengths, including ultra‐low‐field MRI. Researchers are also investigating hyperpolarized probes and nanostructured carriers to enhance relaxometric sensitivity and specificity [67, 68].

A particularly promising, though less explored, area is environmental science. NMR relaxometry has demonstrated potential in the noninvasive analysis of soils, hydration states in porous geological media, and transport of contaminants [69, 70, 71, 72, 73, 74, 75, 76, 77, 78]. The sensitivity of relaxation to molecular interactions allows the detection of subtle changes in soil organic matter, pore water dynamics, and mineral–organic interfaces. FFC‐NMR, for example, has been used to probe hydration shells and bound water in soil matrices, providing a mechanistic understanding of water retention and availability [79, 80]. Moreover, this technique was also applied to provide new parameters to quantify soil erosion with the aim to provide the best conditions for soil remediation and qualification [81, 82, 83, 84, 85, 86].

Recent studies have also explored the detection of microplastics in aqueous environments using low‐field TD‐NMR, exploiting differences in relaxation behavior induced by hydrophobic contaminants [54, 87]. Additionally, relaxometry—as well as MRI and TD‐NMR—is being tested for monitoring industrial and municipal wastewater, offering real‐time feedback on viscosity and solute composition without requiring chemical reagents [88, 89, 90]. These developments point toward the broader integration of NMR relaxometry in sustainable monitoring systems, including precision agriculture and climate‐sensitive soil diagnostics.

From a technological perspective, ongoing miniaturization and integration efforts are likely to enhance the accessibility of relaxometry. Portable or even hand‐held relaxometers are being prototyped for point‐of‐care diagnostics, industrial fieldwork, and environmental sampling [91, 92, 93]. The combination of AI‐driven analysis, Bayesian inference, and machine learning algorithms is streamlining the interpretation of multicomponent relaxation profiles, especially in nonideal and heterogeneous systems [17, 28, 94].

Initiatives such as the European FC‐RELAX project [33] aim to unify many of these threads, developing new hardware platforms and data science tools to support applications in medicine, soft matter, and environmental science.

Looking ahead, NMR relaxometry appears poised to expand its role in multiscale, multidomain characterization, offering both a fundamental understanding of dynamic processes and practical tools for diagnostics, monitoring, and materials design.

## 7 Final Remarks

NMR relaxometry has matured into a robust and versatile analytical approach, capable of probing molecular motion, structure, and interactions across diverse systems and scientific domains. From its origins in the foundational studies of nuclear relaxation to the sophisticated field‐cycling and low‐field instruments available today, the technique has consistently broadened its methodological scope and practical relevance.

The complementary strengths of TD‐NMR and FFC‐NMR offer researchers a flexible and powerful toolbox: TD‐NMR provides rapid, scalable measurements suitable for industrial and soft matter applications, while FFC NMR enables frequency‐resolved characterization of complex dynamics in heterogeneous environments. Continued advances in instrumentation, magnetic field stability, and data analysis methods—including regularized inversion and AI‐assisted processing—are propelling the field forward.

Recent developments have shown that relaxometry is not confined to traditional domains such as material science and biophysics. Emerging fields—including biomedical diagnostics, environmental surveillance, and smart materials engineering—are beginning to adopt relaxometric strategies, driven by advances in miniaturized hardware, portable systems, and real‐time data analytics. Innovative approaches such as ultrafast Laplace NMR and the application of hyperpolarization techniques, as critically reviewed by Telkki [11], offer promising solutions to long‐standing limitations in sensitivity and time efficiency. Although these strategies are currently limited to selected proof‐of‐concept studies, they represent a meaningful shift toward high‐resolution, single‐scan experiments and enhanced accessibility of relaxometric data. However, it is worth noting that the pedagogical review by Telkki [11], while comprehensive in its theoretical scope, does not provide quantitative benchmarks regarding experimental durations, instrumentation costs, or reproducibility under real‐world conditions. Nor does it offer a comparative assessment between conventional NMRD methodologies and ultrafast or hyperpolarized approaches. Furthermore, despite the exciting potential of hyperpolarization strategies, their application to Laplace NMR remains largely prospective, with practical demonstrations still relatively limited.

Several recent studies by Stapf and coworkers [95, 96, 97] have attempted to bridge this gap by combining dynamic nuclear polarization (DNP) with FFC and Laplace NMR protocols, exploring applications beyond proton nuclei—including ^2^H, ^13^C, and ^7^Li—and proposing workflows for enhanced sensitivity and molecular specificity. These works demonstrate the feasibility of coupling hyperpolarization and field‐cycling for dynamic studies, but they often remain constrained to controlled environments, and challenges related to radical‐induced relaxation artifacts, sample stability, and technical complexity still hinder their broader deployment.

Other contributions, such as those by Tickner et al. [98], and Ahola et al. [99], have focused on ultrafast multidimensional Laplace NMR using spatial encoding. While the results are compelling in terms of acquisition speed and spectral resolution, issues of calibration, reproducibility, and sample heterogeneity are not comprehensively addressed. Moreover, there is limited discussion of how these methods compare—in terms of diagnostic power and robustness—with more established relaxometric protocols.

Nonetheless, challenges persist. Sensitivity limitations, the ill‐posed nature of multiexponential analysis, and the absence of standardized acquisition protocols continue to constrain broader adoption. Moreover, regulatory hurdles and limited commercial availability of FFC instruments have hampered clinical translation. Addressing these barriers will require collaborative innovation across physics, engineering, computational science, and domain‐specific applications.

The future of NMR relaxometry lies in its ability to integrate dynamically with multimodal technologies—combining relaxometry with diffusion, spectroscopy, and imaging—and to operate seamlessly across field strengths, from ultra‐low to high field. With ongoing advances in compact design, cloud‐connected analysis, and field‐deployable instrumentation, relaxometry is poised to evolve from a specialized technique into a mainstream analytical platform.

In this perspective, NMR relaxometry is not only refining our understanding of molecular dynamics but is also emerging as a bridge between fundamental science and real‐world solutions in medicine, sustainability, and industry.

## Data Availability

The data that support the findings of this study are available from the corresponding author upon reasonable request.
